# The Prevalence and Clinical Characteristics of *TECTA*-Associated Autosomal Dominant Hearing Loss

**DOI:** 10.3390/genes10100744

**Published:** 2019-09-24

**Authors:** Rika Yasukawa, Hideaki Moteki, Shin-ya Nishio, Kotaro Ishikawa, Satoko Abe, Yohei Honkura, Misako Hyogo, Ryota Mihashi, Tetsuo Ikezono, Tomoko Shintani, Noriko Ogasawara, Kyoko Shirai, Hiroshi Yoshihashi, Takashi Ishino, Koshi Otsuki, Tsukasa Ito, Kazuma Sugahara, Shin-ichi Usami

**Affiliations:** 1 Department of Otorhinolaryngology, Shinshu University School of Medicine, 3-1-1 Asahi, Matsumoto 390-8621, Japan; yasukawar@shinshu-u.ac.jp (R.Y.); moteki@shinshu-u.ac.jp (H.M.); nishio@shinshu-u.ac.jp (S.-y.N.); 2 Department of Hearing Implant Sciences, Shinshu University School of Medicine, 3-1-1 Asahi, Matsumoto 390-8621, Japan; 3 Department of Otolaryngology, National Rehabilitation Center for Persons with Disabilities, 4-1 Namiki, Tokorozawa 359-8555, Japan; ishikawa-kotaro@rehab.go.jp; 4 Department of Otorhinolaryngology, Toranomon Hospital, 2-2-2 Toranomon, Tokyo 105-0001, Japan; abe3387@ybb.ne.jp; 5 Department of Otolaryngology-Head and Neck Surgery, Tohoku University School of Medicine, 1-1 Seiryomachi, Sendai 980-0872, Japan; y-honkura@ktb.biglobe.ne.jp; 6 Department of Otolaryngology-Head and Neck Surgery, Kyoto Prefectural University of Medicine, 465 Kagii-cho, Kyoto 602-8566, Japan; mihyogo@koto.kpu-m.ac.jp; 7 Department of Otolaryngology-Head and Neck Surgery, Kurume University School of Medicine, 67 Asahi-machi, Kurume 830-0011, Japan; mihashi_ryouta@med.kurume-u.ac.jp; 8 Department of Otorhinolaryngology, Saitama Medical University, 38 Morohongo, Moroyama 350-0451, Japan; ikez@saitama-med.ac.jp; 9 Department of Microbiology, Sapporo Medical University School of Medicine, South-1, West-17, Sapporo 060-8556, Japan; shinto211@gmail.com (T.S.); ogasawara.n@sapmed.ac.jp (N.O.); 10 Department of Otorhinolaryngology, Sapporo Medical University School of Medicine, South-1, West-17, Sapporo 060-556, Japan; 11 Department of Otorhinolaryngology-Head and Neck Surgery, Tokyo Medical University, 6-7-1 Nishi-shinjuku, Tokyo 160-0023, Japan; anko.anko25@gmail.com; 12 Department of Medical Genetics, Tokyo Metropolitan Children’s Medical Center, 2-8-29 Musashidai, Tokyo 183-8561, Japan; hiroshi_yoshihashi@tmhp.jp; 13 Department of Otorhinolaryngology, Head and Neck Surgery, Hiroshima University Hospital, 1-2-3 Kasumi, Hiroshima 734-0037, Japan; tishino@hiroshima-u.ac.jp; 14 Department of Otolaryngology, Fukushima Medical University, 1 Hikarigaoka, Fukushima 960-1295, Japan; kotsuki@fmu.ac.jp; 15 Department of Otolaryngology, Head and Neck Surgery, Yamagata University Faculty of Medicine, 2-2-2 Iida-nishi, Yamagata 990-9585, Japan; tuito@med.id.yamagata-u.ac.jp; 16 Department of Otolaryngology, Yamaguchi University Graduate School of Medicine, 1-1-1 Minamikogushi, Ube City 755-8505, Japan; kazuma@yamaguchi-u.ac.jp

**Keywords:** *TECTA*, non-syndromic hearing loss, DFNA8/12, autosomal dominant, prevalence, genotype-phenotype correlation, hearing progression, haplotype analysis

## Abstract

*TECTA* is well known as a causative gene for autosomal dominant mid-frequency hearing loss observed in various populations. In this study, we performed next-generation sequencing analysis of a large Japanese hearing loss cohort, including eight hundred and twelve (812) subjects from unrelated autosomal dominant hearing loss families, to estimate the prevalence and phenotype-genotype correlations in patients with *TECTA* mutations. The prevalence of *TECTA* mutations in Japanese autosomal dominant sensorineural hearing loss families was found to be 3.2%. With regard to the type of hearing loss, the patients with mutations in the nidogen-like domain or ZA domain of *TECTA* showed varied audiograms. However, most of the patients with mutations in the ZP domain showed mid-frequency hearing loss. The rate of hearing deterioration in *TECTA*-associated hearing loss patients and in the normal hearing Japanese control population were the same and regression lines for each group were parallel. We carried out haplotype analysis for four families which had one recurring missense variant, c.5597C>T (p.Thr1866Met). Our results revealed four different haplotypes, suggesting that this mutation occurred independently in each family. In conclusion, *TECTA* variants represent the second largest cause of autosomal dominant sensorineural hearing loss in Japan. The hearing loss progression observed in the patients with *TECTA* mutations might reflect presbycusis. The c.5597C>T mutation occurred in a mutational hot spot and is observed in many ethnic populations.

## 1. Introduction

Hearing loss (HL) is one of the most common sensory disorders. Congenital HL, in particular, affects one in 500–600 newborns and it is speculated that about 60% of cases are caused by gene mutations [1]. Currently, it is considered that approximately 120 genes are associated with sensorineural hearing loss (SNHL) [2], with non-syndromic HL accounting for 70% of SNHL. Among them, autosomal recessive (AR) inheritance accounts for approximately 75% and autosomal dominant (AD) inheritance constitutes approximately 15–20% of cases. Autosomal dominant sensorineural hearing loss (ADSNHL) tends to be post-lingual and progressive HL [3]. Some causative genes are characterized by typical HL patterns affecting only specific frequencies. Distinct mutations in one of the causative genes for ADSNHL, *TECTA* (DFNA8/12), are well known to be associated with mid-frequency HL, while other mutations are involved in high frequency HL. All kinds of mutations are found among various populations. *TECTA* is also a known genetic cause of autosomal recessive HL (DFNB21); however, most of the *TECTA*-associated HL is ADSNHL. It comprises 23 exons, and encodes a 2155 amino acid protein, α-tectorin, which is non-collagenous glycoprotein that constitutes a major component of the tectorial membrane in the cochlea [4,5]. The tectorial membrane lies over the cochlear hair cells and is critical for both the mechanical amplification of acoustic stimulation by the outer hair cells and for its transmission to the inner hair cells, which are the genuine sensory cells [6]. Previous studies have reported that *TECTA*-associated ADSNHL showed characteristic mid- or high-frequency HL depending on the position of the mutations [5,7].

Recently, next-generation sequencing (NGS) has become commonly used for the genetic screening of SNHL. Although hundreds or thousands of variants may be identified by NGS, it is difficult to determine which variants are pathogenic. It is particularly difficult to prioritize potential causal variants within a family with AD inheritance in which only one particular variant leads to the production of the trait. Therefore, we should consider not only NGS analysis results but also the phenotypes of the subjects.

In this study, we (1) elucidated the prevalence of HL caused by *TECTA* mutations in Japanese ADSNHL patients, (2) examined the genotype-phenotype correlations for each domain, (3) analyzed the rate of HL deterioration for *TECTA*-associated HL patients, and (4) carried out haplotype analysis for one variant identified in four unrelated ADSNHL families to confirm whether the mutation occurred in a mutational hotspot or whether it was a founder mutation. 

## 2. Materials and Methods 

### 2.1. Subjects

In this study, we enrolled eight hundred and twelve (812) Japanese subjects (age range: 0–86 years, mean age: 37.1 years) from unrelated ADHL families. This study cohort was taken from 67 otolaryngology clinics across Japan between June 2000 and May 2017. All subjects in this study suffered from non-syndromic HL without any other associated symptoms. A written informed consent was obtained from each proband and their family members prior to participation in this study. This study was approved by the Shinshu University Human Genetic Analysis Ethical Committee, as well as by the ethics committee of each participating institute. The study was conducted in accordance with the Declaration of Helsinki, with the protocol approved by the Ethics Committee of the Shinshu University School of Medicine No. 387, 4 September 2012 and No. 576, 2 May 2017. Clinical information was obtained for each proband and relatives from medical charts. In this study, we collected the following data: (1) pure-tone audiograms, behavioral audiometry, or auditory steady state responses (ASSR); (2) medical history, including onset of HL, progression, and episodes of vertigo; and (3) temporal bone imaging (computed tomography and/or magnetic resonance) if available.

Pure-tone average (PTA) was calculated by air conduction pure-tone audiometry or the ASSR or behavioral audiometry average threshold in the four frequencies (0.5, 1, 2, and 4 kHz). Hearing levels were classified based on the PTA of the better hearing ear: Normal hearing <20 dB; mild HL, 21–40 dB; moderate HL, 41–70 dB; severe HL, 71–95 dB; and profound HL >95 dB. We also calculated the mean hearing level of: (1) mid-frequency; 0.5–2 kHz, and (2) high-frequency; 4–8 kHz. Type of HL was classified based on the following: High-frequency HL, (2) − (1) ≥ +10 dB; flat type HL, (2) − (1) < ±10 dB; and mid-frequency HL, (1) − (2) ≥ +10 dB. 

### 2.2. Genetic Analysis and Pathogenic Interpretation

In this study, we used amplicon re-sequencing for 68 genes previously reported as genetic causes of non-syndromic hereditary HL (Table S1). In brief, amplicon libraries were prepared using an Ion AmpliSeq^TM^ Custom Panel (ThermoFisher Scientific, Waltham, MA, USA), and Ion AmpliSeq v2 plus kit (ThermoFisher Scientific) in accordance with the manufacturer’s instructions. After amplicon library preparation, emulsion PCR, and next-generation sequencing were performed according to the manufacturer’s protocol with Ion 200 sequencing kit (ThermoFisher Scientific) and Ion PGM sequencer (ThermoFisher Scientific) or Ion HiQ chef Kit (ThermoFisher Scientific) and Ion Proton sequencer (ThermoFisher Scientific). The detailed protocol has been described elsewhere [8].

The fastq files obtained were mapped against the human genome (build GRCh37/hg19) using the Torrent Mapping Alignment Program and the variants, including the SNVs, insertions, and deletions, were detected by Torrent Variant Caller plug-in software. The effects of each variant on the protein were analyzed using ANNOVAR software [9]. The variants affecting amino acid sequences (missense, nonsense, insertion/deletion, and splicing variants) were selected from the identified variants. The selected variants were then also filtered as less than 1% of (1) the 1000 genome database [10], (2) the 6500 exome variants [11], (3) the Human Genetic Variation Database (dataset for 1208 Japanese exome variants) [12], and (4) the 333 in-house Japanese normal hearing controls. To estimate the pathogenicity of missense variants, functional prediction software, included in the ANNOVAR software (Sorting Intolerant from Tolerant (SIFT) [13], Polymorphism Phenotyping (PolyPhen2) [14], Likelihood Ratio Test (LRT) [15], Mutation Taster, [16] and Mutation Assessor [17]) were used. Direct sequencing was used to confirm the candidate variants identified through the analysis pipeline. Segregation analysis of family members was also performed by direct sequencing. 

The pathogenicity of the identified variants was evaluated in accordance with the American College of Medical Genetics (ACMG) standards and guidelines [18]. This system classified variants into five categories; pathogenic, likely pathogenic, uncertain significance, likely benign, and benign based on the various evidence. In addition, we referred to Inter Var when we evaluated variants [19]. A combined annotation dependent depletion (CADD) was also utilized to prioritize potential causal variants [20]. 

### 2.3. Haplotype Analysis of the c.5597C>T Variant

We conducted haplotype analysis for the c.5597C>T (p.Thr1866Met) variant identified in four unrelated ADSNHL families. The haplotypes within the 1Mb region surrounding position c.5597 were analyzed using a set of 11 single nucleotide polymorphisms (SNPs) (three sites upstream and eight sites downstream). To select the SNPs for haplotype analysis, Tag SNPs were searched by SNPinfo Web server of the National Institute of Environmental Health Sciences with the Hap Map JPN data set [21]. This analysis was performed using the direct sequencing method. 

Data Availability Statement: The sequencing data are available in the DDBJ databank of Japan (Accession number: JGAS00000000201).

## 3. Results

### 3.1. The Prevalence of TECTA Mutations in Japanese ADSNHL Patients

We identified 32 variants in 35 out of 812 probands with ADSNHL. As shown in Table 1 and Table 2, 26 variants were novel, and six variants were previously reported as causing ADSNHL. Of the 26 novel variants, 25 were missense and one was nonsense. The six previously reported variants were as follows; p.Asp197Asn, p.Thr562Met, p.His1400Tyr, p.Pro1791Arg, p.Thr1866Met, and p.Arg1890Cys [5,22,23,24]. 

**Figure 1:**
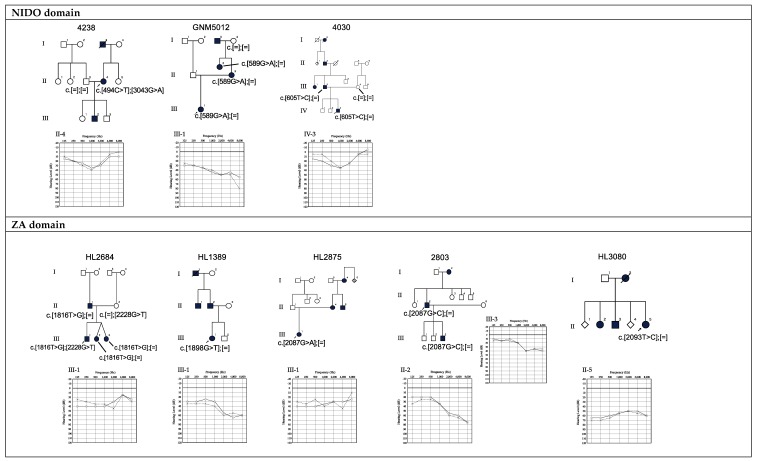

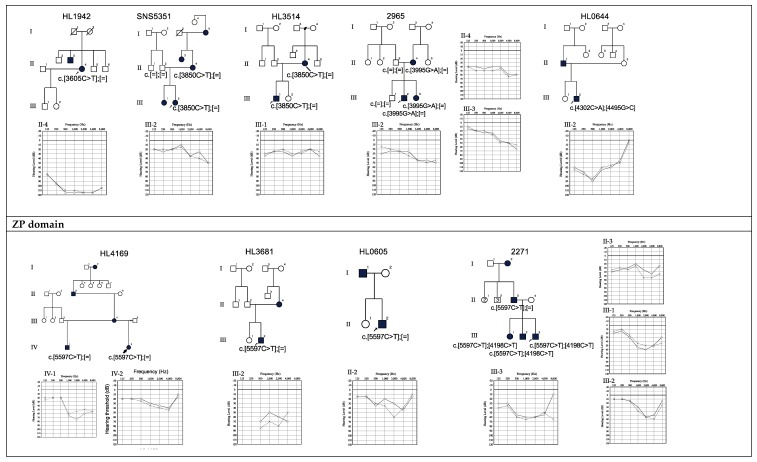

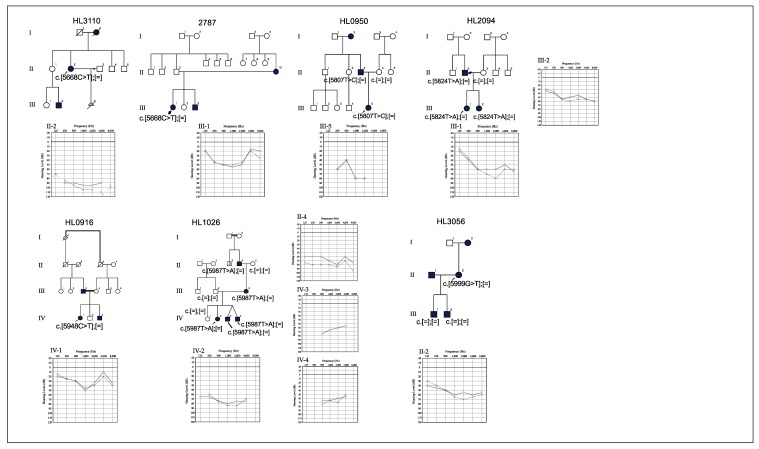

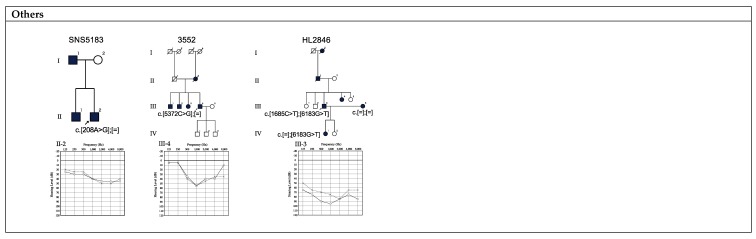
Pedigree and audiograms for each family with *TECTA* variants listed in Table 1. Arrows indicate the probands in each family. Genetic findings for each individual tested are noted in the pedigree. NIDO: nidogen-like domain, ZA: zonahesin-like domain, ZP: zona pellucida domain. Circle and solid line: right ear hearing threshold, X-mark and dotted line: left ear hearing threshold.

We next categorized these variants based on the ACMG criteria and Inter Var [18,19]. Then, we compared the CADD Phred scores for each variant with the larger value of the minor allele frequency (MAF) in the ExAC database [25] or in the ToMMo 3.5KJPN database (Tohoku University Tohoku Medical Megabank Organization, Sendai, JPN) [26] to estimate the pathogenicity, as shown in the scatter plot in Figure S1. We further selected the candidate variants as follows; (1) VUS variants (indicated as blue points in Figure S1) identified in 10 probands were less likely to cause HL, thus we removed these variants from further analysis. (2) We removed the variants with high MAF and/or low CADD scores (under the dotted-line in Figure S1). (3) We removed the one nonsense variant c.4302C>A (p.Tyr1434Ter) as a cause for DFNA8/12. Disease causing mechanism for *TECTA* -associated ADSNHL was thought to be a dominant-negative effect resulting from missense mutations [22,27]. Therefore, it is unlikely that this nonsense mutation is causative for ADSNHL. In addition, the proband (HL0644) with this mutation also carried another variant in the *TECTA* gene; c.4955G>C (p.Asp1499His), which was categorized as VUS, suggesting autosomal recessive inheritance (DFNB21). Finally, we defined the remaining 22 variants as likely causative variants (Table 1). The pedigree and audiograms for each patient are shown in Figure 1 and the variants are listed in Table 1. The *in silico* prediction software scores for novel missense variants are shown in Table S2. 

According to the above results, the prevalence of *TECTA* mutations in Japanese ADSNHL families was considered to be 3.2% (26/812 probands). Furthermore, when we limited our analysis to ‘Likely pathogenic’ variants, it was considered to be 1.8% (15/812 probands). 

Among the identified variants, c.5597C>T was recurrent and identified in four unrelated ADSNHL families. Interestingly, the c.3995G>A variant identified in this study caused a p.Cys1332Tyr amino acid change. Kim et al. have reported the c.3995G>T variant as pathogenic, which despite having a different nucleotide change, resulted in the same amino acid change [28]. 

### 3.2. Genotype-Phenotype Correlation

**Figure 2:**
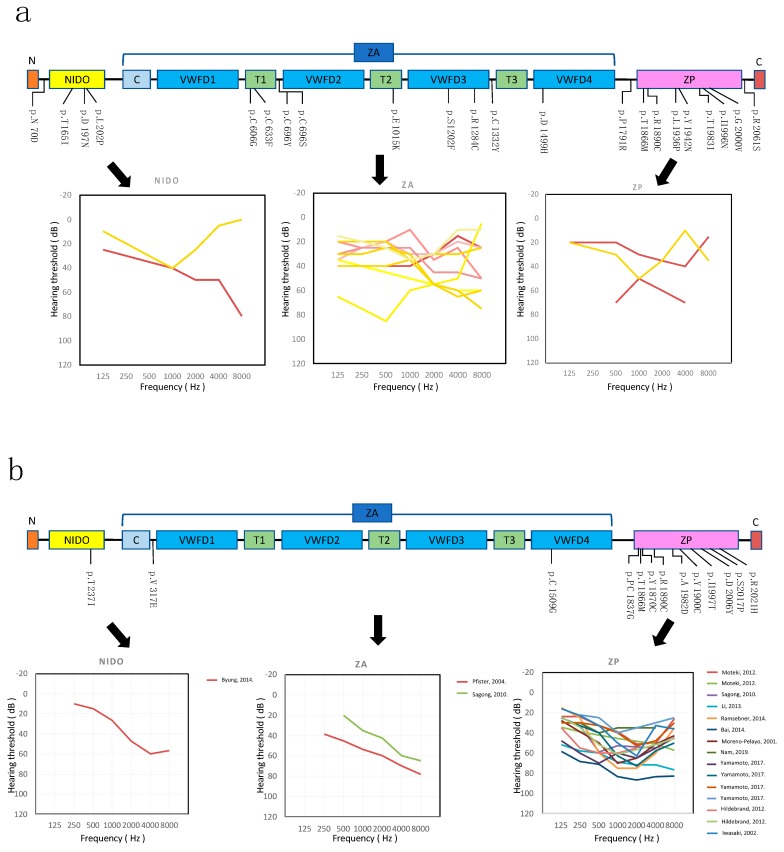
(**a**) Domain structure of α-tectorin and the overlapping audiograms from the better hearing ear for the patients with each domain variant. Yellow lines indicate the candidate VUS variants, and red lines indicate the likely pathogenic variants. (**b**) The overlapping audiograms for the patients that were reported variants of *TECTA* in previously. NIDO: Nidogen-like domain, ZA: Zonahesin-like domain, C: Von Willebrand factor C domain, T(*n*): Trypsin inhibitor-like domain (number), VWD: Von Willebrand factor D domain, ZP: Zona pellucida domain.

α-tectorin has three major components: (1) a nidogen-like (NIDO) domain; (2) a large zonadhesin (ZA) domain containing three trypsin inhibitor-like (TIL) cysteine-rich domains, a von Willebrand factor type C (vWFC) and four von Willebrand factor type D (vWFD) domains; and (3) a zona pellucida (ZP) domain [29]. According to previous reports, mutations affecting the ZP domain are significantly associated with mid-frequency SNHL, whereas mutations in the ZA domain are associated with high-frequency SNHL [5,7]. Figure 2a shows a schema of the α-tectorin domains, and overlapping pure-tone audiograms of individuals corresponding to the position of each domain. 

In the NIDO domain, three variants, c.494C>T, c.589G>A and c.605T>C, were found in one family each. One variant, c.589G>A, led to high-frequency SNHL. The other two variants, c.494C>T and c.605T>C, led to mid-frequency SNHL. 

In the ZA domain, the audiograms were varied, with mid-frequency HL observed in 6 subjects, flat-type HL in six subjects and high-frequency HL in eight subjects (Figure 2a). 

In the ZP domain, seven of 12 variants were associated with mid-frequency HL, and four variants were associated with flat HL. The severity of HL varied among the patients, however nine out of 11 cases had mild-moderate HL (Figure 2a). 

### 3.3. Relationship between Age and Hearing Levels

**Figure 3:**
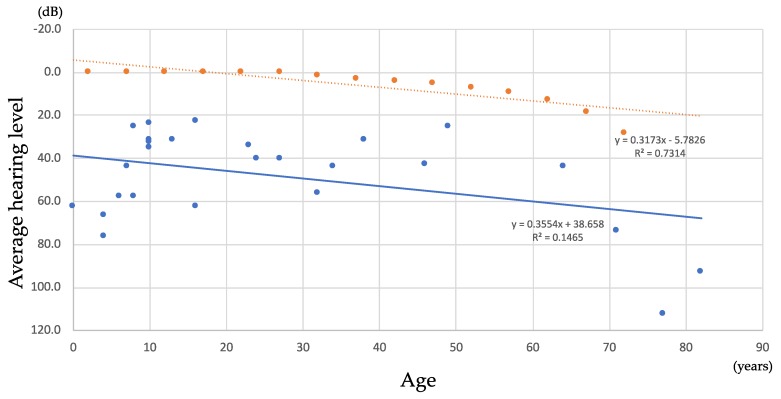
Detailed progression analysis of HL deterioration for patients with *TECTA* mutations. Lines indicate the linear regression for each group. Blue dots indicate the pure-tone average (PTA) of each patient with *TECTA* variants. Red dots indicating the PTA for Japanese normal hearing control population (Tsuiki et al. Audiology Japan 2003, 46, 235–240, in Japanese).

To estimate the rate of hearing deterioration, we compared the age and hearing levels of 26 probands. As shown in Figure 3, each blue point indicated the average hearing level (0.5–4K Hz) obtained at the time of blood sampling. In contrast, the red points are the average hearing level in the Japanese normal hearing population (age: 35–74 y). As a result, the rate of hearing deterioration in both groups was found to be the same and the regression lines for each group were parallel. Thus, hearing deterioration in *TECTA* patients was age-related and may not be accelerated by the gene mutation.

### 3.4. The c.5597C>T (p.Thr1866Met) Variant in the ZP Domain

One missense variant, c.5597C>T (p.Thr1866Met), in the ZP domain was identified in four unrelated families. The audiograms showed flat or mid-frequency HL, and were similar to the previously reported phenotype for variants located in the ZP domain [7]. 

We carried out haplotype analysis for these four families to determine whether this variant arose in a mutational hot spot or was a founder mutation. Table 3 shows the haplotype patterns within the 1-Mb region surrounding this variant.

The different haplotypes observed suggest that this mutation occurred independently in each family. Thus, we suggest that c.5597C>T (p.Thr1866Met) arose from a mutational hot spot. 

## 4. Discussion

In this study, we examined the prevalence of *TECTA* mutations in the Japanese ADSNHL population, and identified 26 *TECTA*-associated HL patients, which account for 3.2% of ADSNHL families. According to previous reports, *TECTA* mutations account for 2.9–5% of all ADSNHL patients [24,30]. Our result is consistent with those previous studies. The findings show that *TECTA* variants represent the second largest cause of ADSNHL in Japan, following *KCNQ4* variants (6.6%) [31]. 

In previous studies, only three mutations within the NIDO domain, c.632T>C (p.Phe211Ser), c.589G>A (p.Asp197Asn), and c.710C>T (p.Thr237Ile), were reported in ADSNHL patients [32]. Furthermore, those cases showed mid- or high-frequency HL (Figure 2b) [32]. In our study, three variants, c.494C>T, c.589G>A, and c.605T>C, were identified in one family each, two of them novel. The patient with known c.589G>A variant showed high-frequency HL, but the other patients (with the novel c.494C>T and c.605T>C variants) showed mid-frequency HL (Figure 2a). The c.589G>A variant was reported previously, but that phenotype was mid-frequency SNHL [32]. Thus, the characteristic phenotype of HL was not observed in the NIDO domain variants. Previously, mutations in the vWFD2-D3 or vWFD4 repeat areas included in the ZA domain have been associated with high-frequency HL (Figure 2b). In this study, we identified 13 variants in the ZA domain region; however, various phenotypes were observed, so we couldn’t identify any trend regarding HL in the ZA domain. 

We also identified 11 variants in the ZP domain and most cases showed mid-frequency HL. This correlation between mutations in the ZP domain and mid-frequency HL has been reported previously (Figure 2b) [9,17,19]. This study is in agreement with the findings of those previous studies. In previous studies, some genes were reported as causative for mid-frequency HL, particularly, *EYA4* (DFNA10), *TECTA* (DFNA8/12), *COL11A2* (DFNA13), *POU4F3* (DFNA15), and *CCDC50* (DFNA44). Among those genes, mutations in *TECTA* are the most frequent cause of mid-frequency HL [1,24,33,34]. Yamamoto et al. reported that pathogenic and possibly pathogenic variants of *TECTA* were found in 6.0% of mid-frequency SNHL patients [35].

In this study, we focused on *TECTA* mutations identified in patients with ADSNHL as most of *TECTA*-associated HL is autosomal dominant and rarely autosomal recessive. The relationship between a domain structure and the inheritance mode of a *TECTA* mutation remains non conclusive. Regarding the type of mutation, missense mutations were predominantly observed in ADSNHL patients, while most loss of function mutations (nonsense, splicing, and frameshift mutations) in *TECTA* were identified in autosomal recessive cases. These results suggest that the mechanism of ADSNHL *TECTA* mutations is presumably dominant negative.

One of the clinical characteristics of the *TECTA*-associated HL was its non-progressive nature [18]. Byung et al. reported that the average HL deterioration rate didn’t exceed 1 dB/year for the NIDO domain variant c.710C>T, p.Thr237Ile [32], and in another study, the p.Arg1890Cys mutation identified in a Dutch ADSNHL family, showed non-progressive, mid-frequency HL [9]. In this study, we analyzed the HL deterioration rate for *TECTA*-associated HL, and found it to be 0.35 dB/year. This HL deterioration rate is comparable to the hearing deterioration rate in a normal hearing control population. A previous report stated that patients with *TECTA*-associated mid-frequency HL might be prone to presbycusis as they are theoretically exposed to a lower level of sound energy as a result of cochlear amplification deficiency [36,37]. However, our results indicated that the hearing loss progression rate was the same as in the control group. Based on these results, we suggest that the mutations in the *TECTA* gene cause functional loss or malformation of the tectorial membrane and inhibit cochlear amplification. As a result of disturbed cochlear amplification, mild to moderate HL is observed. The HL progression (0.3 dB/year) observed in patients with *TECTA* mutations appears to reflect presbycusis, as the HL deterioration rate was comparable with that of normal hearing controls, suggesting that *TECTA* mutations do not accelerate HL deterioration.

In this study, one missense variant, c.5597C>T (p.Thr1866Met), in the ZP domain was detected in four unrelated families. This mutation was also reported in different populations including Americans, Spanish, and Koreans [22]. Hildebrand et al. performed haplotype analysis of the mutation found in the American and Spanish populations and found that they carried different haplotypes suggesting a unique founder effect could be identified in each population [22]. In this study, we also performed haplotype analysis for four families with this mutation and showed they carried different haplotypes even though all of them are Japanese. The results of this study and previous reports suggested that the c.5597C>T mutation arose in a mutational hot spot and occurs regardless of population.

## 5. Conclusions

The prevalence of *TECTA* mutations in Japanese ADSNHL families is estimated as 3.2% (26/812 probands). Furthermore, when we limited our analysis to probands with ‘Likely pathogenic’ or ‘Pathogenic’ variants, it was found to be 1.8% (15/812 probands). In the NIDO and ZA domain regions, various types of HL were observed, so we couldn’t identify any trends. In the ZP domain, most cases showed mid-frequency HL. This result is in agreement with the findings of previous studies. The HL progression observed in the patients with *TECTA* mutations might reflect presbycusis, as the HL deterioration rate was comparable with that of normal hearing controls. A *TECTA* mutation itself is considered not to accelerate the HL deterioration. The recurrent c.5597C>T mutation might have arisen in a mutational hot spot and can be observed in many ethnic populations.

## Figures and Tables

**Table 1:** Summary of the clinical features and information of the likely pathogenic and candidate variants classified as VUS identified in this study.

No.	Sample ID	Gender	Onset Age	Age at Genetic Testing	Fluctuation	Progression	Base Change	AA Change	Domain	Type of HL	Severity	CADD Phred	ACMG Criteria	ACMG Category	MAF in ExAC	MAF in ToMMo	Reference
1	SNS5183	M	16	24	Yes	No	c.208A>G	p.N70D		Flat	Mild	24.0	VUS	PM2, PP3	-	-	This study
2	4238	F	Unknown	49	No	No	c.494C>T	p.T165I	NIDO	Mid	Mild	32.0	VUS	PM1, PM2, PP3	0.0000331	0.0004	This study
3	GNM5012	F	5	7	Unknown	Yes	c.589G>A	p.D197N	NIDO	High	Moderate	27.8	Likely pathogenic	PM1, PM2, PP1, PP3	-	-	Hildebrand, 2011 [22].
4	4030	M	0	10	No	No	c.605T>C	p.L202P	NIDO	Mid	Mild	27.0	Likely pathogenic	PM1, PM2, PP1, PP3	-	-	This study
5	HL2684	F	6	10	No	Yes	c.1816T>G	p.C606G	ZA (TIL1)	Mid	Mild	22.3	Likely pathogenic	PM1, PM2, PP1, PP3	-	-	This study
6	HL1389	F	6	34	No	No	c.1898G>T	p.C633F	ZA (TIL1)	High	Moderate	27.6	VUS	PM1, PM2, PP3	-	-	This study
7	HL2875	F	7	23	Yes	No	c.2087G>A	p.C696Y	ZA	Flat	Mild	26.7	VUS	PM1, PM2, PP3	-	-	This study
8	2803	M	25	46	No	No	c.2087G>C	p.C696S	ZA	High	Moderate	25.6	VUS	PM1,PM2,PP3	-	-	This study
9	4238	F	Unknown	49	No	No	c.3043G>A	p.E1015K	ZA (TIL2)	Mid	Mild	26.4	VUS	PM1,PM2,PP3	0.0000165	0.0004	This study
10	HL1942	F	59	77	No	Yes	c.3605C>T	p.S1202F	ZA (VWD3)	Flat	Profound	24.1	VUS	PM1, PM2, PP3, BS2	0.0000741	0.0001	This study
11	SNS5351	F	12	16	No	No	c.3850C>T	p.R1284C	ZA (VWD3)	High	Mild	26.1	Likely pathogenic	PM1, PM2, PM7, PP3	-	-	This study
12	HL3514	M	6	8	No	No	c.3850C>T	p.R1284C	ZA (VWD3)	Flat	Mild	26.1	Likely pathogenic	PM1, PM2, PM7, PP3	-	-	This study
13	2965	M	6	10	No	No	c.3995G>A	p.C1332Y	ZA	High	Mild	24.4	Likely pathogenic	PM1, PM2, PP1, PP3	-	-	This study
14	HL0644	M	0	16	No	Yes	c.4495G>C	p.D1499H	ZA (VWD4)	Mid	Moderate	29.7	VUS	PM1, PM2, PP3, BS2	-	0.0006	This study
15	3552	M	62	64	Yes	Yes	c.5372C>G	p.P1791R		Mid	Moderate	22.5	VUS	PM2, PP5, BP4	0.0002	-	Hildebrand, 2011 [22].
16	HL4169	F	6	13	No	No	c.5597C>T	p.T1866M	ZP	Flat	Mild	35.0	Likely pathogenic	PM1, PM2, PM7, PP3, PP5	0.00000824	-	Sagong, 2010 [23].
17	HL3681	M	0	0	No	No	c.5597C>T	p.T1866M	ZP	Mid	Moderate	35.0	Likely pathogenic	PM1, PM2, PM7, PP3, PP5	0.00000824	-	Sagong, 2010 [23].
18	HL0605	M	0	10	No	No	c.5597C>T	p.T1866M	ZP	Mid	Mild	35.0	Likely pathogenic	PM1, PM2, PM7, PP3, PP5	0.00000824	-	Sagong, 2010 [23].
19	2271	M	Unknown	6	Unknown	Unknown	c.5597C>T	p.T1866M	ZP	Mid	Moderate	35.0	Likely pathogenic	PM1, PM2, PM7, PP3, PP5	0.00000824	-	Sagong, 2010 [23].
20	HL3110	F	Unknown	82	No	Yes	c.5668C>T	p.R1890C	ZP	Flat	Severe	34.0	Likely pathogenic	PM1, PM2, PP3, PP5	-	-	Plantinga, 2006 [5].
21	2787	F	15	27	Unknown	Yes	c.5668C>T	p.R1890C	ZP	Mid	Mild	34.0	Likely pathogenic	PM1, PM2, PP3, PP5	-	-	Plantinga, 2006 [5].
22	HL0950	F	3	4	No	No	c.5807T>C	p.L1936P	ZP	unspecified	Moderate	25.4	Likely pathogenic	PM1, PM2, PP1, PP3	-	-	This study
23	HL2094	F	3	8	No	No	c.5824T>A	p.Y1942N	ZP	Mid	Moderate	32.0	Likely pathogenic	PM1, PM2, PP1, PP3	-	-	This study
24	HL0916	F	6	38	No	No	c.5948C>T	p.T1983I	ZP	Mid	Mild	33.0	VUS	PM1, PM2, PP3	-	-	This study
25	HL1026	F	0	4	No	No	c.5987T>A	p.I1996N	ZP	Mid	Severe	34.0	Likely pathogenic	PM1, PM2, PP1, PP3	-	-	This study
26	HL3056	F	4	32	Yes	Yes	c.5999G>T	p.G2000V	ZP	Flat	Moderate	26.2	VUS	PM1, PM2, PP3	-	-	This study
27	HL2846	M	2	71	No	No	c.6183G>T	p.R2061S		Mid	Severe	24.4	VUS	PM2, PP1, PP3	-	-	This study

**Table 2:** Summary of the clinical features and information of the plausible benign VUS and likely benign variants identified in this study.

No.	Sample ID	Gender	Onset Age	Age at Genetic Testing	Fluctuation	Progression	Base Change	AA Change	Domain	Type of HL	Severity	CADD Phred	ACMG Criteria	ACMG Category	MAF in ExAC	MAF in ToMMo	Reference
1	HL0150	M	Unknown	60	No	Yes	c.842T>C	p.V281A	ZA (VWC)	High	Moderate	10.5	VUS	PM1, PM2, BP4	-	-	This study
2	SNS5496	F	50	57	No	Yes	c.1049G>A	p.R350Q	ZA (VWD1)	Flat	Mild	25.9	VUS *	PM1, PM2, PP3	0.00000824	-	This study
3	HL0133	F	0	2	No	No	c.1049G>A	p.R350Q	ZA (VWD1)	Flat	Moderate	25.9	VUS *	PM1, PM2, PP3, BP4	0.00000824	-	This study
4	HL0770	F	0	33	No	Yes	c.1424C>T	p.P475L	ZA (VWD1)	Mid	Severe	23.4	VUS *	PM1, PM2, PP3, BS4, BP5	0.00000824	-	This study
5	HL2846	M	2	71	No	No	c.1685C>T	p.T562M	ZA	Mid	Severe	25.6	VUS *	PM1, PP3, PP5	0.000099	0.0001	Hildebrand, 2011 [22].
6	HL3080	F	60	73	No	Yes	c.2093T>C	p.V698A	ZA	Flat	Moderate	14.1	VUS	PM1, PM2, BP4	-	0.0001	This study
7	HL2684	F	6	10	No	Yes	c.2228G>T	p.C743F	ZA (VWD2)	Mid	Mild	24.4	VUS *	PM1, PM2, PP3, BS2, BS4	-	0.0003	This study
8	HL1091	F	5	42	No	Yes	c.2228G>T	p.C743F	ZA (VWD2)	High	Profound	24.4	VUS *	PM1, PM2, PP3, BS2, BS4	-	0.0003	This study
9	HL4176	F	0	45	No	Yes	c.2228G>T	p.C743F	ZA (VWD2)	Flat	Profound	24.4	VUS *	PM1, PM2, PP3, BS2, BS4	-	0.0003	This study
10	HL1937	F	45	77	No	Yes	c.3556C>T	p.R1186W	ZA (VWD3)	High	Severe	33.0	VUS *	PM1, PP3, BS1, BS4	0.0009	0.0001	This study
11	2271	M	Unknown	6	Unknown	Unknown	c.4198C>T	p.H1400Y	ZA (TIL3)	Mid	Moderate	25.0	VUS *	PM1, PP3, BS2	0.0002	0.0016	Moteki,2012 [24].
12	HL0644	M	0	16	No	Yes	c.4302C>A	p.Y1434X	ZA	Mid	Moderate	36.0	VUS *	PM2	-	-	This study
13	HL0280	M	0	9	No	No	c.5908G>A	p.A1970T	ZP	Flat	Mild	13.5	Likely Benign	BS2, BP4	0.0000906	0.001	This study

Nucleotide and protein positions of *TECTA* variants are according to RefSeq: NM_005422. M: Male, F: Female, AA: Amino acid, NIDO: Nidogen-like domain, ZA: Zonahesin-like domain, VWC: Von Willebrand factor C domain, TIL: Trypsin inhibitor-like domain, VWD: Von Willebrand factor D domain, ZP: Zona pellucida domain, MAF: Minor allele frequency. * ACMG category indicates the evidence for each variant classification.

**Table 3:** The haplotypes around the *TECTA*: c.5597C>T mutation identified from four families.

Distance from the c.5597C>T Mutation (bp)	Marker	Fm1		Fm2		Fm3								Fm4				Allele Frequency (HapMap-JPT)			
		HL3681		HL0605		2271		* 6		* 7		* 5		HL4169		* 4					
						A	U	A	U	A	U	A	U	A	U	A	U				
429809	rs752979	T	C	T	C	T	T	T	T	T	T	C	C	T	C	T	C	C	0.46	T	0.54
315340	rs4430518	T	G	T	T	G	G	T	T	T	T	T	G	T	T	T	T	G	0.36	T	0.64
236767	rs4936565	G	G	A	G	A	G	A	A	A	G	A	G	G	G	G	G	A	0.34	G	0.66
0	c.5597C>T	-		-		-		-		-		-		-		-					
589752	rs7941422	G	G	G	G	G	A	G	G	G	G	G	G	G	G	G	G	A	0.17	G	0.83
649183	rs621360	T	C	T	T	T	C	T	T	T	T	T	T	C	T	C	C	C	0.24	T	0.76
822346	rs2852833	T	T	G	T	G	T	G	G	G	G	G	G	G	G	G	G	G	0.63	T	0.37
1008518	rs515449	G	G	A	A	A	G	A	G	A	A	A	A	A	A	A	A	A	0.65	G	0.35
1089754	rs489877	A	A	A	C	A	A	A	A	A	A	A	A	A	C	A	A	A	0.72	C	0.28
1145749	rs528219	C	C	C	T	C	C	C	C	C	C	C	C	C	T	C	C	C	0.69	T	0.31
1292838	rs3906964	A	A	G	G	G	A	G	A	G	A	G	G	G	G	G	A	A	0.26	G	0.74
1495732	rs7117842	T	T	T	T	T	C	T	C	T	C	T	C	T	C	T	C	C	0.37	T	0.63

Red columns show the same region shared by unrelated families. Blue columns indicate different regions among families. Fm: Family, A: Putative affected allele, U: Putative unaffected allele. * (number): The family member with a *TECTA* mutation which is same variant to the each proband in Figure 1.

## Supplementary Materials

The following are available online at https://www.mdpi.com/2073-4425/10/10/744/s1, Figure S1: Scatter plot of each identified variant’s CADD score and higher MAF value in ToMMo 3.5kJPN or ExAC0.3., Table S1: The 68 target genes for hearing loss., Table S2: The *in silico* prediction scores for novel variants in this study.
