# Supplementary material for: The Prevalence and Clinical Characteristics of TECTA-Associated Autosomal Dominant Hearing Loss
Source: Genes (Basel). 2019 Sep 24;10(10):744. doi: 10.3390/genes10100744 (PMC6826443; doi:10.3390/genes10100744)
Supplement: Supplementary file 1 [file genes-10-00744-s001.zip › genes-575821-supplementary-final/Table S2.pdf]

Table S2. The in silico prediction scores for novel variants in this study.

Sample ID	Base Change	AA Change	Domein	SIFT	PP2_ HumDiv	PP2_ HumVar	LRT	MutTaster	MutAssessor	FATHMM	MetaSVM	LR
SNS5183	c.208A>G	p.N70D		D (0.912)	D (0.637)	D (0.725)	D (0.843)	D (0.466)	M (0.832)	D (0.823)	D (0.928)	D (0.912)
4238	c.494C>T	p.T165I	NIDO	D (0.721)	D (0.764)	D (0.875)	D (0.629)	D (0.588)	H (0.944)	D (0.822)	D (0.950)	D (0.936)
4030	c.605T>C	p.L202P	NIDO	D (0.912)	D (0.764)	D (0.749)	D (0.629)	D (0.810)	H (0.949)	T (0.739)	D (0.896)	D (0.870)
HL2684	c.1816T>G	p.C606G	ZA (TIL1)	T (0.301)	D (0.899)	D (0.875)	D (0.843)	D (0.588)	H (0.975)	D (0.982)	D (0.982)	D (0.994)
HL1389	c.1898G>T	p.C633F	ZA (TIL1)	D (0.912)	D (0.899)	D (0.916)	D (0.843)	D (0.810)	H (0.990)	D (0.957)	D (0.992)	D (0.988)
HL2875	c.2087G>A	p.C696Y	ZA	D (0.912)	D (0.899)	D (0.916)	D (0.843)	D (0.588)	H (0.941)	T (0.270)	T (0.711)	T (0.624)
2803	c.2087G>C	p.C696S	ZA	D (0.912)	D (0.899)	D (0.875)	D (0.843)	D (0.588)	H (0.941)	T (0.266)	T (0.711)	T (0.624)
4238	c.3043G>A	p.E1015K	ZA (TIL2)	D (0.654)	D (0.764)	D (0.832)	D (0.843)	D (0.548)	M (0.852)	D (0.911)	D (0.965)	D (0.959)
HL1942	c.3605C>T	p.S1202F	ZA (VWD3)	D (0.506)	P (0.420)	B (0.409)	D (0.466)	D (0.343)	N (0.088)	T (0.606)	T (0.585)	T (0.420)
SNS5351	c.3850C>T	p.R1284C	ZA (VWD3)	D (0.446)	P (0.515)	P (0.443)	D (0.485)	D (0.462)	M (0.612)	T (0.379)	T (0.193)	T (0.373)

HL3514	c.3850C>T	p.R1284C	ZA (VWD3)	D (0.447)	P (0.515)	P (0.443)	D (0.485)	D (0.462)	M (0.613)	T (0.379)	T (0.193)	T (0.373)
2965	c.3995G>A	p.C1332Y	ZA	D (0.721)	D (0.571)	P (0.521)	D (0.843)	D (0.810)	H (0.963)	D (0.838)	D (0.941)	D (0.922)
HL0644	c.4495G>C	p.D1499H	ZA (VWD4)	D (0.912)	D (0.899)	D (0.971)	D (0.843)	D (0.810)	M (0.791)	T (0.787)	D (0.898)	D (0.887)
HL0950	c.5807T>C	p.L1936P	ZP	T (0.240)	D (0.899)	D (0.916)	D (0.843)	D (0.810)	L (0.520)	D (0.856)	D (0.905)	D (0.909)
HL2094	c.5824T>A	p.Y1942N	ZP	D (0.912)	D (0.764)	D (0.875)	D (0.843)	D (0.588)	M (0.712)	D (0.841)	D (0.936)	D (0.925)
HL0916	c.5948C>T	p.T1983I	ZP	D (0.912)	D (0.764)	D (0.875)	D (0.843)	D (0.588)	M (0.758)	D (0.860)	D (0.937)	D (0.936)
HL1026	c.5987T>A	p.I1996N	ZP	D (0.784)	D (0.764)	D (0.875)	D (0.843)	D (0.588)	M (0.646)	D (0.842)	D (0.932)	D (0.923)
HL3056	c.5999G>T	p.G2000V	ZP	D (0.531)	D (0.899)	D (0.971)	D (0.843)	D (0.810)	L (0.520)	D (0.950)	D (0.975)	D (0.975)
HL2846	c.6183G>T	p.R2061S		D (0.555)	D (0.577)	D (0.697)	D (0.843)	D (0.453)	M (0.640)	T (0.488)	T (0.613)	T (0.571)

PP2: PolyPhen2, MutTaser: Mutation Taser, MutAssessor: Mutation Assessor, D: probably damaging or deleterious or disease causing, P: possibly damaging, T: tolerated, B: benign, H: high, M: medium, L: low, N: neutral
