# Supplementary material for: The Prevalence and Clinical Characteristics of TECTA-Associated Autosomal Dominant Hearing Loss
Source: Genes (Basel). 2019 Sep 24;10(10):744. doi: 10.3390/genes10100744 (PMC6826443; doi:10.3390/genes10100744)
Supplement: Supplementary file 1 [file genes-10-00744-s001.zip › genes-575821-supplementary-final/TECTA_supplementary files- figure s1+ table s1.pdf]

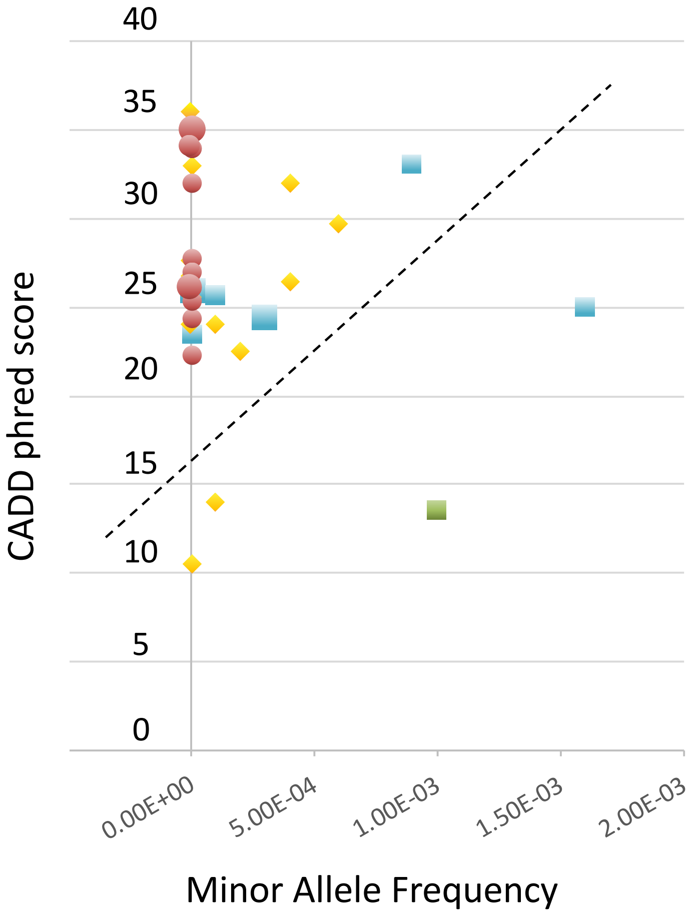


**Figure S1.** Scatter plot of each identified variant's CADD score and higher MAF values in ToMMo 3.5kJPN or ExAC0.3. The color of each point corresponds to the interpretation based on the ACMG criteria in Table1. Red indicates likely pathogenic variants. Yellow indicates candidate variants classified as VUS identified in this study. Blue indicates the VUS variants we removed from the analysis. Green indicates a likely benign variant. The dotted-line separates two groups; the putative benign variants with a high MAF and/or low CADD score, and the candidate variants with a low MAF and/or high CADD score.

**Table S1.** The 68 target genes for hearing loss.

| Number | Locus Symbol | Gene Symbol | Gene ID | Transcript Variant |
|---|---|---|---|---|
| 1 | DFNA1 | *DIAPH1* | NM_005219 | NM_001079812 |
| 2 | DFNA2 | *KCNQ4* | NM_004700 | NM_172163 |
| 3 | DFNA2 | *GJB3* | NM_024009 | NM_001005752 |
| 4 | DFNA3 | *GJB6* | NM_006783 | NM_001110219<br>NM_001110220<br>NM_001110221 |
| 5 | DFNA4 | *MYH14* | NM_024729 | NM_001145809.<br>NM_001077186 |
| 6 | DFNA5 | *DFNA5* | NM_004403 | NM_001127454<br>NM_001127453 |
| 7 | DFNA6/14/38 | *WFS1* | NM_006005 | NM_001145853 |
| 8 | DFNA8/12/DFNB21 | *TECTA* | NM_005422 | - |
| 9 | DFNA9/31 | *COCH* | NM_004086 | NM_001135058 |
| 10 | DFNA10 | *EYA4* | NM_004100 | NM_172103<br>NM_172105 |
| 11 | DFNA11/DFNB2/USH1B | *MYO7A* | NM_000260 | NM_001127179<br>NM_001127180 |
| 12 | DFNA13/DFNB53/STL3 | *COL11A2* | NM_080680 | NM_080679<br>NM_080681<br>NM_001163771 |
| 13 | DFNA15 | *POU4F3* | NM_002700 | - |
| 14 | DFNA17 | *MYH9* | NM_002473 | - |
| 15 | DFNA20/DFNA26 | *ACTG1* | NM_001614 | NM_001199954 |
| 16 | DFNA22/DFNB37 | *MYO6* | NM_004999 | - |
| 17 | DFNA25 | *SLC17A8* | NM_139319 | NM_001145288 |
| 18 | DFNA28 | *GRHL2* | NM_024915 | - |
| 19 | DFNA36/DFNB7/11 | *TMC1* | NM_138691 | - |
| 20 | DFNA40 | *CRYM* | NM_001888 | NM_001014444 |
| 21 | DFNA44 | *CCDC50* | NM_178335 | NM_174908 |
| 22 | DFNA48 | *MYO1A* | NM_005379 | - |

| | | | | |
|---|---|---|---|---|
| 23 | DFNA50 | *MIRN96* | | |
| 24 | DFNA51 | *TJP2* | NM_001170414 | NM_004817<br>NM_201629<br>NM_001170630<br>NM_001170415<br>NM_001170416 |
| 25 | DFNA64 | *DIABLO* | NM_019887 | NM_138929 |
| 26 | | *CEACAM16* | NM_001039213 | - |
| 27 | DFNB1/DFNA3 | *GJB2* | NM_004004 | - |
| 28 | DFNB3 | *MYO15A* | NM_016239 | - |
| 29 | DFNB4/Pendred | *SLC26A4* | NM_000441 | - |
| 30 | DFNB6 | *TMIE* | NM_147196 | - |
| 31 | DFNB8/10 | *TMPRSS3* | NM_024022 | NM_032405 |
| 32 | DFNB9 | *OTOF* | NM_194248 | NM_194322<br>NM_194323<br>NM_004802 |
| 33 | DFNB12/USH1D | *CDH23* | NM_022124 | NM_001171930<br>NM_001171931<br>NM_001171932<br>NM_001171933<br>NM_001171934<br>NM_001171936<br>NM_052836 |
| 34 | DFNB15/72/95 | *GIPC3* | NM_133261 | - |
| 35 | DFNB16 | *STRC* | NM_153700 | - |
| 36 | DFNB18/USH1C | *USH1C* | NM_153676 | NM_005709 |
| 37 | DFNB22 | *OTOA* | NM_144672 | NM_001161683<br>NM_170664 |
| 38 | DFNB23/USH1F | *PCDH15* | NM_033056 | NM_001142763<br>NM_001142764<br>NM_001142765<br>NM_001142766 |

| | | | | |
|---|---|---|---|---|
| | | | | NM_001142767<br>NM_001142769<br>NM_001142770<br>NM_001142771<br>NM_001142772<br>NM_001142773 |
| 39 | DFNB24 | *RDX* | NM_002906 | - |
| 40 | DFNB25 | *GRXCR1* | NM_001080476 | - |
| 41 | DFNB28 | *TRIOBP* | NM_007032 | NM_138632<br>NM_001039141 |
| 42 | DFNB29 | *CLDN14* | NM_144492 | NM_001146077<br>NM_001146078<br>NM_001146079<br>NM_012130 |
| 43 | DFNB30 | *MYO3A* | NM_017433 | - |
| 44 | DFNB31/USH2D | *WHRN* | NM_015404 | NM_001083885<br>NM_001173425 |
| 45 | DFNB35 | *ESRRB* | NM_004452 | - |
| 46 | DFNB36 | *ESPN* | NM_031475 | - |
| 47 | DFNB39 | *HGF* | NM_000601 | NM_001010931<br>NM_001010932<br>NM_001010933<br>NM_001010934 |
| 48 | DFNB42 | *ILDR1* | NM_001199799 | NM_001199800<br>NM_175924 |
| 49 | DFNB48 | *CIB2* | NM_006383 | NM_001271888<br>NM_001271889 |
| 50 | DFNB49 | *MARVELD2* | NM_001038603 | NM_001244734 |
| 51 | DFNB59 | *DFNB59* | NM_001042702 | - |
| 52 | DFNB61 | *SLC26A5* | NM_206883 | NM_001167962 |

| | | | | |
|---|---|---|---|---|
| | | | | NM_206884<br>NM_206885<br>NM_198999 |
| 53 | DFNB63 | *LRTOMT* | NM_001145307 | NM_001145308<br>NM_001205138<br>NM_145309 |
| 54 | DFNB66/67 | *LHFPL5* | NM_182548 | - |
| 55 | DFNB70 | *PNPT1* | NM_033109 | - |
| 56 | DFNB74 | *MSRB3* | NM_198080 | NM_001031679<br>NM_001193460<br>NM_001193461 |
| 57 | DFNB77 | *LOXHD1* | NM_144612 | NM_001173129<br>NM_001145472<br>NM_001145473 |
| 58 | DFNB79 | *TPRN* | NM_001128228 | - |
| 59 | DFNB82 | *GPSM2* | NM_013296 | - |
| 60 | DFNB84 | *PTPRQ* | NM_001145026 | - |
| 61 | DFNB89 | *KARS* | NM_005548 | NM_001130089 |
| 62 | DFNB91 | *SERPINB6* | NM_004568 | NM_001195291 |
| 63 | DFNB93 | *CABP2* | NM_016366 | - |
| 64 | USH2A | *USH2A* | NM_007123 | NM_206933 |
| 65 | DFNX1 | *PRPS1* | NM_002764 | NM_001204402 |
| 66 | DFNX2 | *POU3F4* | NM_000307 | - |
| 67 | DFNX4 | *SMPX* | NM_014332 | - |
| 68 | DFNX6 | *COL4A6* | NM_001847 | NM_033641 |
